# Changing patterns in the medical care of refugees with HIV from Ukraine

**DOI:** 10.1007/s15010-026-02867-y

**Published:** 2026-07-03

**Authors:** Gerrit Ahrenstorf, Alexandra Dopfer-Jablonka, Christine Knuth, Theresa Graalmann, Thea Thiele, Luca Wolf, Rene Abu Isneineh, Felix C. Ringshausen, Hortense Slevogt, Torsten Witte, Georg M. N. Behrens

**Affiliations:** 1 Department of Rheumatology and Immunology, Hannover Medical School, Carl-Neuberg-Str. 1, 30625 Hannover, Germany; 2 Deutsches Zentrum für Infektionsforschung (DZIF), partner site Hannover- Braunschweig, Hannover, Germany; 3 Department of Gastroenterology, Hepatology, Infectious Diseases and Endocrinology, Hannover Medical School, Carl-Neuberg-Str. 1, 30625 Hannover, Germany; 4 Department of Respiratory Medicine and Infectious Diseases, Hannover Medical School, Carl-Neuberg-Str. 1, 30625 Hannover, Germany; 5 Biomedical Research in End-stage and Obstructive Lung disease (BREATH), Deutsches Zentrum für Lungenforschung (DZL), Hannover, Germany; 6 European Reference Network on Rare and Complex Respiratory Diseases (ERN-LUNG), Frankfurt, Germany; 7 Cluster of Excellence RESIST (EXC 2155), Hannover Medical School, Hannover, Germany

**Keywords:** HIV infection, Ukraine, Refugees, Forced migration, Antiretroviral therapy, Virological failure, Hepatitis co-infection, Germany

## Abstract

Since February 2022, the war in Ukraine has led to large-scale displacement, including people living with HIV (PLWH). Early reports described predominantly virologically suppressed individuals with preserved immune function. Data on refugees with HIV presenting during later phases of the war are limited. We conducted a retrospective single-center study including adult refugees with HIV from Ukraine presenting to a tertiary care center in Germany. Paticipants were grouped by time of presentation: Cohort 1 (March–December 2022) and Cohort 2 (January 2023–February 2024). Clinical, immunological, and virological parameters were compared between cohorts. A total of 86 individuals were included (Cohort 1: *n* = 46; Cohort 2: *n* = 40). Participants in cohort 2 more frequently received their first HIV diagnosis after presentation in Germany, with no previously documented HIV diagnosis in Ukraine or elsewhere, compared with cohort 1 (67% vs. 5%; *p* < 0.0001). Participants in Cohort 2 more often presented with detectable HIV RNA (65% vs. 29%; *p* < 0.0001) and advanced immunodeficiency (CD4 < 200/µL: 28% vs. 9%; *p* < 0.05). Among individuals with prior HIV diagnosis, virological failure was more common in Cohort 2 (50% vs. 14%; *p* = 0.006). AIDS-defining illnesses occurred more frequently in Cohort 2 (*p* = 0.005). Serological evidence of HBV and HCV infection was high in both cohorts. Refugees with HIV from Ukraine presenting during later phases of the war exhibit more advanced disease and poorer virological control compared with earlier arrivals. These findings suggest increasing disruption of HIV care over time and highlight the need for low-threshold access to testing, comprehensive screening, and rapid initiation of antiretroviral therapy in this population.

## Background

Since February 2022, the Russian invasion of Ukraine has resulted in the largest displacement of civilians in Europe since World War II. By mid-2023, approximately 5.3 million individuals had fled Ukraine to seek refuge in other European countries [1, 2]. Given the comparatively high prevalence of HIV infection in Ukraine prior to the conflict, a substantial proportion of these refugees are people living with HIV (PLWH) [3, 4].

Several observational cohort studies from European host countries have described the clinical characteristics of PLWH who left Ukraine during the early phase of the war. These reports consistently indicate that most individuals were receiving effective antiretroviral therapy (ART) before displacement and presented with preserved immunological status, commonly defined by CD4 T-cell counts above 200 cells/µL and suppressed plasma HIV RNA levels below the limit of detection. This observation has been attributed to sustained access to HIV care in urban regions of Ukraine before the invasion and to the preferential mobility of clinically stable PLWH during the initial period of displacement [5–7].

In contrast, limited data are available on PLWH who migrated at later stages of the conflict. Ongoing disruption of healthcare services, reduced availability of antiretroviral medication, and cumulative psychosocial stressors may have disproportionately affected individuals with advanced HIV disease or suboptimal treatment adherence. Consequently, it may be anticipated that PLWH presenting to care after prolonged exposure to wartime conditions exhibit poorer immunological and virological parameters [8, 9].

We hypothesized that PLWH from Ukraine who presented to healthcare services in host countries after the first year of the war differ clinically from those who migrated earlier. To test this hypothesis, we compared two cohorts of Ukrainian PLWH presenting to HIV care during different phases of the conflict. The objective of this study was to evaluate differences in immunological and virological status and to assess potential implications for the organization of HIV care for displaced populations.

### Participants and methods

All adult individuals with confirmed HIV infection and a history of forced displacement from Ukraine who presented to our specialized outpatient HIV clinic were eligible for inclusion in this study.  PLWH were consecutively enrolled without exclusion criteria in order to minimize selection bias and to reflect routine clinical practice.

Participants were stratified into two cohorts according to the date of their initial presentation to our outpatient clinic. Individuals who presented between 15 March 2022 and 30 December 2022 were assigned to cohort 1, representing refugees who sought medical care during the early phase of the war.  PLWH who presented between 1 January 2023 and 22 February 2024 were allocated to cohort 2, representing those who arrived after a prolonged period of conflict.

Demographic and clinical data were obtained through standardized medical history interviews conducted by trained physicians using a structured questionnaire. All interviews were performed in the Russian language to ensure adequate comprehension and accuracy of the collected information. Collected variables included age, sex, prior HIV diagnosis, previous antiretroviral treatment, and time since departure from Ukraine.

Laboratory parameters were extracted from routine clinical records and included CD4 T-cell counts and plasma HIV RNA levels at the time of first presentation. HIV infection was confirmed using standard diagnostic algorithms, including fourth-generation HIV antigen/antibody screening assays followed by confirmatory immunoassays and HIV-RNA testing, as documented in the hospital laboratory information system.

HBV screening included HBsAg, anti-HBc, and anti-HBs testing. In PLWH with positive HBsAg or clinical suspicion of chronic HBV infection, HBV DNA testing was performed. HCV screening was performed using anti-HCV antibody testing. In participants with positive anti-HCV serology, confirmatory HCV RNA testing was performed to assess active infection. All laboratory analyses were performed as part of standard-of-care diagnostics in certified laboratories. Data were anonymized prior to analysis to ensure confidentiality.

The study was conducted in accordance with local ethical standards and the principles of the Declaration of Helsinki. This retrospective study was based exclusively on routinely collected clinical data obtained during standard care. No additional interviews or study-specific data collection procedures were performed for research purposes.

According to local ethical regulations and institutional review board approval, written informed consent was waived for this retrospective analysis of pseudonymised routine clinical data. Data obtained from standardized intake interviews and the hospital information system were linked using pseudonymised participant identifiers and analysed in compliance with applicable data protection regulations. Relevant variables were subsequently extracted into a separate pseudonymised research dataset for analysis.

Statistical analyses were performed using GraphPad Prism software. Continuous variables are presented as median and interquartile range (IQR) and were compared using the Mann–Whitney U test. Categorical variables were analysed using Fisher’s exact test or chi-square test as appropriate. A p value < 0.05 was considered statistically significant.

## Results

A total of 86 refugees with confirmed HIV infection were included in the analysis. Of these, 46 individuals presented during the first year of the war (cohort 1), whereas 40  persons sought care during the second and third years of the conflict (cohort 2). In cohort 1, 41  participants (89%) were female and 5 (11%) were male. In cohort 2, 27  participants (75%) were female and 13 (25%) were male. The median age was 41 years (interquartile range [IQR], 34–45) in cohort 1 and 45 years (IQR, 35–46) in cohort 2.

In cohort 1, heterosexual contact was the most commonly presumed mode of HIV transmission and was reported in 20 cases (44%), while intravenous drug use (IVDU) was documented in 9 individuals (20%). In 17 cases (37%), the mode of transmission remained unknown. No cases of men who have sex with men (MSM) were reported in this cohort.

In cohort 2, heterosexual contact was reported in 19 individuals (48%), IVDU in 11 cases (28%), and MSM transmission in one case (2%), whereas the mode of transmission was unknown in 9 individuals (22%) (table 1).

PLWH in cohort 1 originated predominantly from Kyiv (*n* = 8), Odesa (*n* = 6), Kryvyi Rih (*n* = 5), Mariupol (*n* = 4), Dnipro (*n* = 4), Kharkiv (*n* = 3), Donetsk (*n* = 3), Chernihiv (*n* = 3), Melitopol (*n* = 2), Mykolaiv (*n* = 2), Lutsk (*n* = 2), Zaporizhzhia (*n* = 2), and Zhytomyr (*n* = 2).

Participants in cohort 2 originated from Kyiv (*n* = 10), Odesa (*n* = 5), Donetsk (*n* = 3), Kharkiv (*n* = 3), Kremenchuk (*n* = 3), Luhansk (*n* = 3), Chernihiv (*n* = 3), and Melitopol (*n* = 3), followed by Mariupol (*n* = 2), Mykolaiv (*n* = 2), and Zaporizhzhia (*n* = 2).

### Duration of HIV infection and timing of diagnosis

The median duration since HIV diagnosis differed significantly between the two cohorts. In cohort 1, HIV infection had been known for a median of 8.0 ± 7.15 years, whereas in cohort 2 the median duration was 3.5 ± 1.5 years (*p* < 0.05). This difference was largely attributable to a substantially higher proportion of newly diagnosed HIV infections in Germany among participants in cohort 2 compared with cohort 1 (67% vs. 11%, respectively; *p* < 0.0001).

Participants were classified as having a previous HIV diagnosis in Ukraine if they reported a known HIV infection prior to displacement and/or provided medical documentation, antiretroviral medication records, or prior treatment history consistent with established HIV care in Ukraine.

Newly diagnosed HIV infection in Germany was defined as first documented HIV diagnosis established after presentation in Germany in individuals without a prior known HIV diagnosis according to medical history and available documentation.

### Immunological status and virological control

Cohort 2 comprised a significantly higher proportion of treatment-naïve individuals with detectable plasma HIV RNA levels compared with cohort 1 (65% vs. 22%; *p* < 0.0001). In addition, advanced immunosuppression, defined as a CD4 T-cell count below 200 cells/µL, was more frequently observed in cohort 2 than in cohort 1 (28% vs. 9%; *p* < 0.05).

**Fig. 1:**
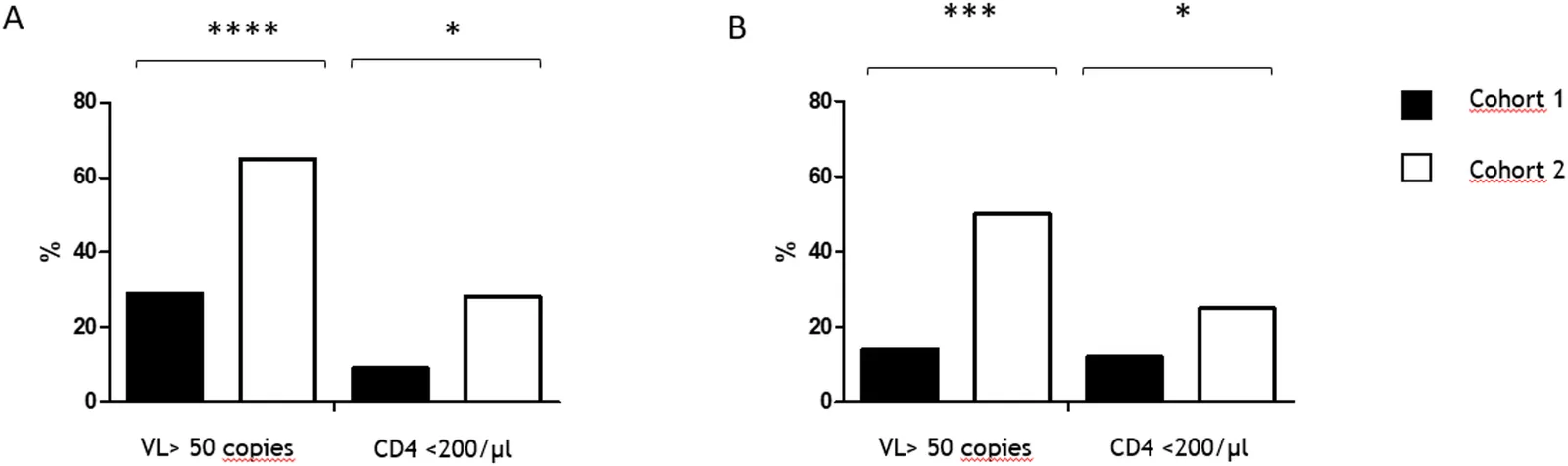
Proportion of refugees living with HIV presenting with detectable plasma HIV RNA and CD4 T-cell counts < 200 cells/µL during the first year of the war (cohort 1) compared with those presenting in 2023 and 2024 (cohort 2). Refugees in cohort 2 exhibited a significantly higher proportion of individuals with detectable HIV RNA and CD4 T-cell counts < 200 cells/µL compared with cohort 1 (panel A). This difference was not solely attributable to the higher proportion of newly diagnosed HIV infections in Germany among participants in cohort 2. When the analysis was restricted to individuals with a previously established HIV diagnosis in Ukraine, cohort 2 still demonstrated a significantly higher proportion of participants with detectable viral load and impaired immunological status compared with cohort 1 (panel B)

Notably, even among individuals whose HIV infection had already been diagnosed in Ukraine prior to displacement, virological control differed significantly between the cohorts. A higher proportion of these individuals in cohort 2 presented with detectable HIV RNA compared with those in cohort 1 (50% vs. 14%; *p* = 0.006). Furthermore, AIDS-defining conditions were significantly more prevalent among refugees in cohort 2 (*p* = 0.005), indicating a higher burden of advanced HIV disease in this group (Fig. 1).

### Antiretroviral therapy

In Cohort 1, the most frequently prescribed antiretroviral regimen (70%) was the fixed-dose combination of tenofovir disoproxil fumarate (TDF), lamivudine (3TC), and dolutegravir (DTG). Alternative regimens included TDF/emtricitabine (FTC)/efavirenz (EFV), abacavir (ABC)/3TC/DTG, TDF/FTC/DTG, and tenofovir alafenamide (TAF)/FTC/bictegravir (BIC).

As the fixed-dose combination of TDF/3TC/DTG is not available in Germany, participantswere offered either continuation of the same regimen using separate tablets (DTG + 3TC/TDF) or a switch to an equivalent single-tablet regimen available in Germany, most commonly TAF/FTC/BIC. The majority of participants  (90%) preferred continuation of their existing regimen using separate tablet formulations.

In Cohort 2, TDF/3TC/DTG remained the most frequently used regimen, although in a lower proportion of individuals (40%). A higher proportion of participants were receiving TAF/FTC/BIC (*n* = 8; 20%). Participants already receiving antiretroviral therapy were likewise offered a switch to available single-tablet regimens, whereas treatment-naïve individuals were preferentially initiated on single-tablet regimens containing TAF/FTC/BIC or on DTG/3TC-based therapy.

In cohort 1, no participant reported previous HIV care in Germany or other European countries prior to presentation at our clinic. In contrast, two individuals in cohort 2 had previously presented to HIV care centres in Germany, and four additional participants had received HIV care in other European countries before attending our institution. In five cases, antiretroviral therapy had already been modified during these interim consultations.

### Hepatitis B virus infection

Serological evidence of past hepatitis B virus (HBV) infection was detected in more than half of the participants in cohort 1 and in 40% of participants in cohort 2. No cases of active replicative HBV infection were identified in either cohort.

### Hepatitis C virus infection

HCV prevalence was assessed at first presentation by anti-HCV antibody testing and confirmatory HCV RNA analysis. Antibodies against hepatitis C virus (HCV) were detected in 21  participants  (46%) in cohort 1 and in 16  participants  (40%) in cohort 2. However, only 10 individuals in cohort 1 and 7 individuals in cohort 2 were aware of their HCV infection based on medical history. Previous antiviral treatment for HCV in Ukraine had been documented in five cases. Detectable HCV RNA was found in 8 participants  (17%) in cohort 1 and in 6 participants  (15%) in cohort 2, indicating ongoing viral replication.

All participants with confirmed chronic HCV infection were offered further evaluation and treatment. All but one participant , who declined therapy, received DAA treatment and achieved sustained virological response (SVR).

## Discussion

In this study, we observed substantial differences in the clinical and virological characteristics of people living with HIV (PLWH) from Ukraine who presented to care during different phases of the ongoing war. Refugees who sought medical attention during the early phase of the conflict were predominantly individuals with a known HIV diagnosis and established antiretroviral therapy (ART) prior to displacement. In contrast, PLHW presenting during the second and third years of the war more frequently received their initial HIV diagnosis in Germany and often exhibited advanced stages of disease, including pronounced immunosuppression and higher rates of detectable plasma HIV RNA.

Notably, even among individuals with a previously established HIV diagnosis in Ukraine, those who presented at later stages of the conflict showed significantly poorer virological control and lower CD4 T-cell counts compared with individuals who migrated earlier. These findings suggest that prolonged exposure to wartime conditions, including disruption of healthcare infrastructure, interruptions in ART supply chains, reduced access to specialized HIV care, and repeated displacement, may have contributed to deterioration of clinical status over time.

Previous reports have similarly described substantial challenges in maintaining HIV care continuity and antiretroviral treatment access during the ongoing war in Ukraine [4, 11].

Importantly, our findings indicate that the clinical profile of displaced PLWH from Ukraine changed substantially as the war progressed. While early refugee cohorts were predominantly characterized by previously diagnosed individuals with established ART and virological suppression, our later cohort increasingly included with newly diagnosed HIV infection, uncontrolled viremia, advanced immunodeficiency, and AIDS-defining conditions. Our data therefore suggest that early cohort studies may not fully reflect the evolving healthcare needs and medical complexity of displaced populations during prolonged armed conflict.

These observations are of particular relevance given previous reports from European host countries, which largely described cohorts of Ukrainian refugees with preserved immune function and effective viral suppression [3–5]. Our findings from Cohort 1 are also consistent with results from the multicentre German ORANGE study presented at the European AIDS Conference (EACS 2023, Warsaw), which included 941 Ukrainian PLWH receiving HIV care in Germany and reported high rates of virological suppression at first presentation as well as frequent antiretroviral treatment modifications after migration (10). These findings appear most comparable to our Cohort 1, which predominantly consisted of PLWH presenting during the first year of the war with previously diagnosed and virologically controlled HIV infection.

While such findings were reassuring during the early phase of displacement, they may not adequately reflect the evolving epidemiological situation among PLWH who remain in or leave Ukraine at later stages of the conflict. Early refugee cohorts may therefore not fully reflect the evolving medical needs and disease burden of displaced populations during prolonged armed conflict.

Our results extend these earlier observations by demonstrating that later refugee cohorts may represent a substantially more vulnerable population with higher medical complexity and greater need for immediate diagnostic and therapeutic interventions.

The high prevalence of hepatitis B and hepatitis C virus co-infections observed in both cohorts further underscores the necessity of comprehensive infectious disease screening in displaced populations from Ukraine. In particular, the substantial proportion of PLWH with previously undiagnosed HCV infection highlights potential gaps in prior access to testing and antiviral treatment. These findings are consistent with epidemiological data indicating an increased burden of viral hepatitis among PLWH in Eastern Europe and reinforce the importance of integrated screening strategies upon arrival in host countries.

From a public health and clinical perspective, our results emphasize the ongoing need for low-threshold, non-discriminatory access to HIV testing and care for refugees from Ukraine. Healthcare providers caring for displaced individuals from Ukraine should remain vigilant for previously undiagnosed or insufficiently treated HIV infection, particularly in PLWH with interrupted healthcare access or unknown treatment history. Rapid linkage to ART and comprehensive infectious disease screening remain essential, as delayed diagnosis and treatment initiation are associated with increased morbidity, mortality, and ongoing transmission risk. Furthermore, continuity of care across borders and improved coordination between healthcare providers in transit and host countries may help mitigate treatment interruptions and improve long-term outcomes (Table 1).

Several limitations must be acknowledged. The retrospective and single-center design restricts the generalizability of our findings. In addition, socioeconomic factors, degree of war-related destruction, and detailed information on internal displacement within Ukraine were not systematically available and could therefore not be included in the analysis. Similarly, detailed data regarding exact duration of treatment interruption prior to presentation were not available for all individuals. Nevertheless, our data suggest that early cohort studies may have underestimated the burden of advanced HIV disease among Ukrainian refugees. The later cohort included in our analysis may more accurately reflect the complex and dynamic clinical realities emerging as the war persists.

In conclusion, PLWH from Ukraine who present to care at later stages of the conflict differ substantially from those who migrated earlier, with higher rates of newly diagnosed and advanced HIV infection as well as frequent viral hepatitis co-infections. These findings highlight the importance of sustained surveillance and adaptable healthcare strategies to address the evolving needs of displaced populations during prolonged humanitarian crises.

Continuous access to healthcare services, including antiretroviral therapy and routine HIV monitoring, has been a key determinant of treatment continuity among Ukrainian refugees in Germany since 2022. Potential changes in healthcare access policies may therefore have important implications for continuity of care and long-term public health outcomes in this vulnerable population.

**Table 1:** Baseline demographic, clinical, immunological, and virological characteristics of refugees living with HIV from Ukraine presenting during the early phase of the war (Cohort 1) and during later phases of the conflict (Cohort 2)

	Cohort 1 (*n* = 46)	Cohort 2 (*n* = 40)	*P* value
Age (median, IQR)	39 (32–45)	45 (35–46)	*n*.s.
Female	41 (89%)	27 (75%)	n.s.
Previous HIV diagnosis	41 (89%)	13 (33%)	*p* < 0.0001
First diagnosis in Germany	5 (11%)	27 (67%)	*p* < 0.0001
Detectable HIV RNA	10 (22%)	26 (65%)	*p* < 0.0001
CD4 < 200/µl	4 (9%)	11 (28%)	*p* < 0.05
Heterosexual transmission	20 (44%)	19 (48%)	n.s.
IVDU	9 (20%)	11 (28%)	n.s.
MSM	0	1 (2%)	n.s.
unknown	17 (33%)	9 (22%)	n.s.
Anti-HBs positive	19 (41%)	16 (40%)	n.s.
Anti-HBc positive	23 (50%)	16 (40%)	n.s.
HBV-DNA	0	0	n.s.
Anti-HCV positive	23 (50%)	16 (40%)	n.s.
HCV-RNA positive	8 (17%)	6 (15%)	n.s.

Continuous variables are presented as median and interquartile range (IQR). Categorical variables are presented as number and percentage

*HBV* hepatitis B virus, *HCV* hepatitis C virus, *IVDU* intravenous drug use

## Data Availability

All data supporting the findings of this study are available within the paper and its Supplementary Information.
